# Self-Directed Learning Readiness and Its Correlation With Academic Performance Among Medical Students at King Saud University: A Cross-Sectional Study

**DOI:** 10.7759/cureus.107962

**Published:** 2026-04-29

**Authors:** Noorah Aldubaib, Basmah Alzahrani, Nada Alobaid, Alanoud Almufarrej, Esraa Alnazzawi, Nouf Alsaigh, Raghad Almashan, Naif A Alfahed, Abdulrahman Almusallam, Mohammed Aldawsari

**Affiliations:** 1 Department of Family and Community Medicine, College of Medicine, King Saud University, King Saud University Medical City, Riyadh, SAU; 2 College of Medicine, King Saud University, Riyadh, SAU

**Keywords:** academic performance, cross sectional study, medical education, medical students, saudi arabia, self-directed learning readiness score (sdlrs), self-directed learning (sdl)

## Abstract

Background: Self-directed learning (SDL) is a fundamental component of modern medical education and is essential for lifelong learning among physicians.

Objective: To assess SDL readiness (SDLR) among medical students at King Saud University (KSU) and examine its association with academic performance.

Methods: A quantitative cross-sectional study was conducted among undergraduate medical students at KSU. A total of 330 students participated. Data were collected using Fisher’s Self-Directed Learning Readiness Scale (SDLRS), a validated 40-item questionnaire. Descriptive statistics were used to summarize SDLR scores, and ordinal logistic regression was performed to assess factors associated with grade point average (GPA).

Results: The mean SDLR score was 150 ± 24.9, indicating moderate-to-high readiness for SDL. Male students constituted 189 (57.3%) and female students 141 (42.7%). A total of 192 (58.2%) reported a GPA of 4.5-5.0. No significant differences were observed across gender or academic year. Higher SDLR scores were significantly correlated with higher GPA categories; however, the effect size was small.

Conclusions: Medical students at KSU demonstrate moderate-to-high readiness for SDL. SDLR scores were positively correlated with academic performance; however, the small effect size suggests limited practical significance. These findings highlight the role of SDL as one of multiple factors influencing academic outcomes. Further longitudinal research is needed to better understand this relationship.

## Introduction

Self-directed learning (SDL) has become a central expectation in contemporary medical education, particularly as physicians are required to continuously update their knowledge, critically appraise evidence, and adapt to rapidly evolving healthcare systems. Rather than representing a simple independent study, SDL encompasses a structured process in which learners identify knowledge gaps, set learning goals, select appropriate resources, and evaluate their own progress. Readiness for SDL, therefore, reflects the extent to which students possess the motivation, self-management skills, and cognitive strategies necessary for effective lifelong learning [1-4].

The importance of SDL has increased further with the integration of digital learning environments, simulation-based training, and technology-enabled healthcare delivery. For example, recent work examining physicians’ engagement with telemedicine in the United Arab Emirates highlights how modern clinical practice requires continuous self-directed adaptation to new tools and systems [5]. These shifts reinforce the need for medical graduates who are capable of independently acquiring and updating knowledge beyond formal curricula.

Previous studies have reported variability in SDL readiness (SDLR) across different educational settings. In Saudi Arabia, early work demonstrated generally adequate SDLR among first-year medical students, while more recent studies have identified differences between preclinical and clinical phases [6,7]. International studies similarly emphasize that SDLR is shaped by contextual factors such as institutional culture, faculty support, and assessment strategies [8,9].

The relationship between SDL and academic performance remains an important area of investigation. Evidence from systematic reviews indicates that SDL may improve learning outcomes, particularly when supported by structured educational approaches [3,4]. However, findings are not entirely consistent, and the extent to which SDLR translates into measurable academic achievement may depend on the surrounding learning environment [10]. We hypothesized that higher SDLR scores would be positively associated with higher grade point average (GPA) categories.

## Materials and methods

Study design

This was a quantitative cross-sectional study conducted at the College of Medicine, King Saud University (KSU), Riyadh, Saudi Arabia.

Study population and sample size

Undergraduate medical students from first to fifth academic years were eligible to participate. Interns and postgraduate trainees were excluded. Participation was voluntary, and incomplete responses were excluded. A total of 330 students were included.

A sample size calculation was performed using the Raosoft sample size calculator (Raosoft Inc., Seattle, WA, USA) assuming a 95% confidence level, a 5% margin of error, and a 50% response distribution. The minimum required sample size was 306 participants. The final sample of 330 students exceeded this requirement, ensuring adequate statistical power for the analysis.

Participants were recruited using a convenience sampling approach through electronic distribution of the questionnaire. Participation was anonymous, and no identifying information was collected.

Study measures

SDLR was assessed using Fisher’s Self-Directed Learning Readiness Scale (SDLRS), a validated 40-item instrument with established reliability and construct validity in health professions education [11,12]. The SDLRS has been widely used in diverse educational settings, including Middle Eastern populations, supporting its applicability in this context [11,12].

Each item is rated on a five-point Likert-type scale, a widely used ordinal response format for measuring attitudes and perceptions in educational research [13]. Total scores were calculated by summing all item responses. SDLRS scores were categorized as low (<132), moderate (132-160), and high (>160) based on previously reported interpretation thresholds [11,12]. Negatively worded items were reverse-coded prior to analysis to ensure consistent scoring direction. Internal consistency of the SDLRS in the current sample was high (Cronbach’s alpha = 0.95). Additional variables included gender, academic year, and self-reported GPA. GPA categories were based on the institutional grading system at KSU.

Ethics statement

Ethical approval was obtained from the Institutional Review Board of KSU (Approval No: E-25-10489). Participation was voluntary, and informed consent was obtained electronically.

Statistical analysis

Data were analyzed using IBM SPSS Statistics for Windows, Version 27 (Released 2019; IBM Corp., Armonk, New York, United States). Descriptive statistics (means, standard deviations, frequencies, and percentages) were used to summarize the data. Normality of continuous variables was assessed using the Shapiro-Wilk test. Comparisons between two groups were performed using t-tests, while comparisons across more than two groups were conducted using a one-way ANOVA. Ordinal logistic regression analysis was conducted to assess factors associated with GPA categories. A p-value < 0.05 was considered statistically significant. 

## Results

A total of 330 medical students participated in the study. Male students constituted 189 (57.3%), while female students accounted for 141 (42.7%). Participants were distributed across all academic years, with the largest proportions in the fourth year (96, 29.1%) and fifth year (114, 34.5%). Academic performance was skewed toward higher GPA categories, with 192 (58.2%) reporting GPA 4.5-5.0 and 100 (30.3%) reporting GPA 4.0-4.49. The demographic and academic characteristics of participants are summarized in Table 1.

**Table 1:** Gender and academic characteristics of medical students (n = 330) GPA: grade point average

Variable	n	%
Male	189	57.3
Female	141	42.7
1st Year	47	14.2
2nd Year	39	11.8
3rd Year	34	10.3
4th Year	96	29.1
5th Year	114	34.5
GPA < 3.0	1	0.3
GPA 3.0-3.40	8	2.4
GPA 3.5-3.99	29	8.8
GPA 4.0-4.49	100	30.3
GPA 4.5-5.0	192	58.2

The overall mean SDLR score was 150 ± 24.9. The SDLRS demonstrated excellent internal consistency in this study (Cronbach’s α = 0.95). The mean SDLR score falls within the moderate-to-high range of the scale, indicating moderate-to-high readiness for SDL. Regarding planning and organization, 221 (67.0%) students reported solving problems using a plan, and 235 (71.2%) reported prioritizing their work. Additionally, 231 (70.0%) reported confidence in independent learning. Comparisons of mean SDLR scores by gender showed no statistically significant difference between male and female students (151.19 ± 21.88 vs. 149.40 ± 28.53; p = 0.536). Similarly, no statistically significant differences were observed across academic years, with mean SDLR scores remaining relatively consistent from first to fifth year (p = 0.428) (Table 2). However, a slight increasing trend in SDLR scores across academic years was observed.

**Table 2:** Comparison of SDLR scores by gender and academic year SDLRS: Self-Directed Learning Readiness. Data are presented as mean ± standard deviation. Statistical significance was set at p < 0.05.

Group	Mean SDLR score ± SD	P-value
Male	151.19 ± 21.88	0.536
Female	149.40 ± 28.53	0.536
1st Year	146.43 ± 27.76	0.428
2nd Year	150.69 ± 27.01	0.428
3rd Year	145.00 ± 25.76	0.428
4th Year	151.83 ± 24.45	0.428
5th Year	152.40 ± 23.04	0.428

Ordinal logistic regression analysis was conducted to assess factors associated with GPA categories. In univariate analysis, male students had significantly lower odds of being in higher GPA categories compared with female students (OR = 0.46, 95% CI: 0.29-0.72, p < 0.001). However, this association was no longer statistically significant after adjustment for other variables in the multivariable model (adjusted OR = 0.67, 95% CI: 0.40-1.09, p = 0.111). The academic year was independently associated with GPA. Compared with first-year students, those in the second through fifth years had lower odds of being in higher GPA categories in both univariate and multivariable analyses, which may reflect differences in grading structures and academic demands across training phases. Importantly, the total SDLR score demonstrated a significant positive association with GPA. Each one-point increase in SDLR score was associated with a slight increase in the odds of being in a higher GPA category in both univariate (OR = 1.02, 95% CI: 1.01-1.03, p < 0.001) and multivariable analyses (adjusted OR = 1.03, 95% CI: 1.02-1.04, p < 0.001) (Table 3). Although statistically significant, the effect size was small (adjusted OR = 1.03), indicating that the SDLR score has a modest association with GPA and may not represent a clinically or educationally meaningful effect independently.

**Table 3:** Ordinal logistic regression analysis for gender, academic year, and total SDLR score factors associated with GPA SDLRS: Self-Directed Learning Readiness Scale; GPA: grade point average; OR: odds ratio; CI: confidence interval; Ref: reference category. Statistical significance at P-value < 0.05.

Variable	Unadjusted OR (95% CI)	P-value	Adjusted OR (95% CI)	P-value
Gender				
Female	Reference			
Male	0.46 (0.29-0.72)	<0.001	0.67 (0.40-1.09)	0.111
Academic Year				
1st Year	Reference			
2nd Year	0.08 (0.02-0.28)	<0.001	0.07 (0.01-0.27)	<0.001
3rd Year	0.06 (0.01-0.20)	<0.001	0.07 (0.01-0.25)	<0.001
4th Year	0.08 (0.02-0.23)	<0.001	0.06 (0.01-0.24)	<0.001
5th Year	0.08 (0.02-0.25)	<0.001	0.07 (0.02-0.24)	<0.001
Total SDLR Score	1.02 (1.01-1.03)	<0.001	1.03 (1.02-1.04)	<0.001

## Discussion

The findings of this study indicate that medical students at KSU generally demonstrate a relatively moderate-to-high readiness for SDL. These findings align with previous literature indicating that health professions students often demonstrate moderate-to-high SDLR in learner-centered curricula [3,4,9]. The lack of statistically significant differences across academic years may reflect relatively stable SDLR across training levels. However, this should not be interpreted as evidence that SDLR is established early in training, as the cross-sectional design does not allow for assessment of developmental progression. This finding may also suggest that SDL competencies do not necessarily increase progressively across academic levels, which is consistent with previous literature highlighting variability in SDL development [9,10]. Our findings are consistent with prior Saudi studies. Soliman and Al-Shaikh reported good SDLR among first-year students, while Mohamed et al. demonstrated variability across training phases [6,7]. Similar international findings emphasize that SDLR may be influenced more by educational context than by academic seniority alone [8,9]. A statistically significant association was observed between SDLR and academic performance. However, this relationship should be interpreted cautiously. Although higher SDLRS scores were correlated with higher GPA categories, the effect size was small (adjusted OR = 1.03), indicating that SDLR contributes only modestly to academic performance. This suggests that SDL is likely one of multiple factors influencing academic outcomes, alongside variables such as prior academic ability, study habits, and assessment structure [14]. While several studies report positive relationships between SDL and academic performance, other studies have demonstrated inconsistent or non-significant associations, highlighting the importance of contextual and curricular factors in shaping this relationship [3,4]. Therefore, SDLR alone may not be a sufficient predictor of academic success. Effective SDL requires structured support rather than complete independence. Previous research emphasizes that SDL is best developed through guided learning strategies, feedback, and appropriate assessment methods [1,15]. Educational approaches such as problem-based learning and structured reflection have been shown to support SDL development when implemented within supportive curricular frameworks [16]. Additionally, the increasing integration of digital learning and telemedicine into healthcare highlights the growing importance of SDL in preparing future physicians for evolving clinical environments [4,5]. These findings provide additional evidence from the Saudi context, where institutional and curricular factors play an important role in shaping SDLR [6-9,14]. Additionally, previous studies have emphasized the importance of valid measurement tools in assessing SDLR among medical students [11,12,17]. Several limitations should be acknowledged. The cross-sectional design limits causal inference, and the study was conducted at a single institution, which may affect generalizability. The use of convenience sampling may introduce selection bias and limit the representativeness of the sample. Additionally, self-reported GPA may introduce reporting bias. Furthermore, although the SDLRS is widely used, it may not fully capture all aspects of self-regulated learning behavior and was not specifically validated for the Saudi context [10-12]. Moreover, potential confounding variables such as prior academic ability, study habits, and teaching quality were not controlled for in this study [14]. Residual confounding cannot be excluded. Future research should explore longitudinal changes in SDLR and examine its association with broader outcomes such as clinical competence and professional development [10,18].

## Conclusions

This study found that medical students at KSU exhibit moderate-to-high readiness for SDL. Higher SDLRS scores were significantly correlated with academic performance; however, the magnitude of this relationship was modest. These findings suggest a potential role of structured SDL strategies within medical education. However, SDLR should be considered as one of multiple factors influencing academic outcomes. Future research should explore longitudinal changes in SDLR and examine its association with broader outcomes such as clinical competence and professional development.
